# Personalising Practice Using Preferences for Meditation Anchor Modality

**DOI:** 10.3389/fpsyg.2018.02521

**Published:** 2018-12-11

**Authors:** Thomas Anderson, Norman A. S. Farb

**Affiliations:** Department of Psychology, University of Toronto, Toronto, ON, Canada

**Keywords:** preference, motivation, heart-rate, personalisation, MBI, mindfulness, meditation, attention

## Abstract

Many people are starting to establish contemplative practices and Mindfulness-Based Interventions have become quite popular. While Mindfulness-Based Interventions positively impact well-being, drop-out and lack of practice-maintenance plagues these interventions. Such adherence issues may reveal a lack of fit between participant partiality for attentional anchors of meditative practice and the intervention’s use of the breath as the anchor of attention. No study had yet compared partiality towards practices using anchors from different sensory modalities (e.g., auditory and visual) thus the present study examined such individual differences, sharing resources on the Open Science Framework^1^. Participants (*N* = 82) engaged 10-min practices within three modalities (somatosensory, auditory, and visual) and partiality towards these meditations was modelled. Partiality differences did exist: 49% preferred the breath, 30% the auditory-phrase, and 21% the visual-image. Pre-practice motivation and anchor-modality predicted partiality while cardiac responses were also positively associated with partiality. Preferences were updated through experience and over half of participants left the experiment partial to a different anchor than their initial meditation-naïve bias. Tangible next-steps are discussed, including integrating additional anchor modalities into existing interventions by offering brief practices with a variety of anchors. Suggestions are made for increasing post-training contact using email-automation to answer central practice-maintenance questions, including whether and which contemplative benefits are predicated on continued practice.

## Introduction

Growing numbers are engaging in contemplative practices to cultivate and maintain well-being. The scientific literature encourages these efforts suggesting that Mindfulness-Based Interventions (MBIs) positively impact well-being measures across a variety of domains (Khoury et al., 2013; Goyal et al., 2014). Benefits may be “gradual, progressive and [requiring] regular practice" (Grossman et al., 2004, 36) thus fostering the establishment and maintenance of daily self-directed contemplative practice is one goal of MBIs.

Unfortunately, approximately 30% of participants in MBIs drop out before completing even half the available sessions (Crane and Williams, 2010; Khoury et al., 2013; Nam and Toneatto, 2016). While attrition rates issues are known, indicators for identifying which participants will drop out are lacking (Dobkin et al., 2012; Hanley et al., 2016; Nam and Toneatto, 2016). In addition to attrition, up to half of course-completing participants skip self-directed practice (Crane et al., 2014), though most research lacks measures addressing this question and factors predicting practice-maintenance have yet to be established (Grossman et al., 2004; Nam and Toneatto, 2016). Failing to establish self-directed practice during a course seems a likely warning sign that practice may be dropped following MBI completion. Failure to establish practice may undermine intervention-related benefits predicated on practice-maintenance (Grossman et al., 2004; Crane et al., 2014) thus more attention should be directed towards identifying initial barriers to establishing practice.

One such barrier may be mismatches between participant preferences and practice style. Practices may vary between how focused the aperture of attention is, often progressing from a single concentrative object such as the breath or sound, before transitioning into a more open monitoring of experience (Lutz et al., 2008). However, in these introductory, focused attention practices, the object of attention itself is often taken for granted, with a focus on the breath or a body area in mindfulness traditions (Kabat-Zinn, 1990), or a subvocalised mantra in Transcendental Meditation (Ospina et al., 2007). Little attention has been paid to whether individuals differ in partiality towards different practice styles. Different practice styles use different attentional anchors and arbitrarily prescribing one anchor-type over others may prevent some participants from establishing an at-home practice. Partiality – the disposition or inclination a person has towards a practice, whether favourable, neutral, or unfavourable – may also change with experience. Partiality may increase when practices promote rewarding psychophysiological states of relaxation or engagement (Shearer et al., 2015) and decrease if aversive states of frustration or confusion arise (Farias and Wikholm, 2016). Partiality may be diminished by difficulty and individuals may be partial to practices that reflect their extant abilities, e.g., composers may be partial to an auditory-anchored practice while gymnasts may favour a somatosensory anchor, such as the breath. Using partiality to inform which practice is prescribed may thereby contribute to establishing and maintaining self-directed contemplative practice (Swift et al., 2011).

### Practice Anchors

Modern MBIs use somatosensory anchors of attention, almost universally beginning with breath-focused awareness to anchor attention. By extension, breath- and body-based practice constitutes the majority of contemplative science research and the clinical and non-clinical benefits of breath-based mindfulness practices are abundantly documented (Khoury et al., 2013; Goyal et al., 2014; Mrazek et al., 2014). These somatosensory-practices are not, however, universally established, maintained, or enjoyed: beyond the drop-out rates discussed above, research also describes adverse reactions to breath-based practices (Anderson et al., 2016; Lindahl et al., 2017).

Investigators have also explored auditory phrase-based practices (e.g., mantra meditation), such as the phrase-based Transcendental Meditation technique (Travis and Shear, 2010). Independent work has also shown effects of silently repeated speech (Berkovich-Ohana et al., 2015) and semantically meaningful phrases are often used in value-based meditations, e.g., compassion/loving-kindness practices (Kristeller and Johnson, 2005). Silently repeated auditory-anchors provoke distinct patterns of neural activation from breath-anchored practices, reducing activity in the anterior cingulate cortex and insular cortex rather than activating them (Fox et al., 2016). With both distinct and overlapping mechanisms of action, whether the choice of anchor is intrinsic to the efficacy of the intervention remains an open question worthy of future research (Schoormans and Nyklíček, 2011; Travis, 2011).

Contemplative practices involving visual stimuli have been far less studied, perhaps due to the complexity of many image-based practices and the religious iconography often employed. Image-based practices may involve complex imagery, such as moving “energy fields” (“qi-gong,” Burke, 2012), or deities in complex scenes (“Vajrayana,” Amihai and Kozhevnikov, 2014). Performing such complex practices in a laboratory may be unrealistically demanding for a novice when compared to simpler anchors like the breath or a short phrase. Similarly, religious iconography runs counter to secular MBI mandates that have allowed them to thrive in Western institutions (Farb, 2014). A simple and secular visual anchor could circumvent these limitations: the plain coloured-disk used as an anchor of attention in one image-based practice (“kasina” practice, Amihai and Kozhevnikov, 2014).

No study has compared partiality for anchors using different sensory modalities (somatosensory, auditory, and visual), though participants differed in their partiality after trying four types of somatosensory-based practices (Burke, 2012). In the broader clinical literature matching partiality for treatment options significantly decreases likelihood of drop-out (Swift et al., 2011), yet partiality-matching is not presently offered in MBIs. While partiality-matching in MBIs may plausibly help decrease drop-out and facilitate practice-establishment, it is not yet know whether partiality for different sensory anchors exists. The current study investigated and revealed partiality differences between three anchors using different sensory modalities: breath-based somatosensory practice, phrase-based auditory practice, and image-based visual practice.

### The Present Study: Partiality and Its Possible Predictors

The present study examined individual differences in partiality for contemplative practice anchors. Participants attempted brief practices within each modality (somatosensory, auditory, and visual) allowing for a characterisation of individual differences in partiality. We attempted to model post-practice partiality as a function of several candidate predictors: instruction-naïve partiality, partiality after reading meditation instructions but before practicing, sensory discriminability, dispositional personality and mindfulness, and physiological response to practice. While there is good reason to expect that partiality could promote adherence (Swift et al., 2011), the present study does not address adherence directly, instead seeking to justify future longitudinal research.

#### Instruction-Naïve and Practice-Naïve Partiality

Partiality is the disposition a person has towards a practice, whether favourable, neutral, or unfavourable. Our most basic hypothesis was that individual differences in partiality between practice anchors exist (H1: Partiality existence). Assuming such partiality would be found, we had a number of hypotheses concerning the prediction of partiality. We hypothesised that, once participants had a chance to read meditation-specific instructions, a higher motivation to engage the specific anchor would predict higher partiality towards that anchor (H1a: Motivation). We also expected participants to begin with some baseline partiality, i.e., partiality existing before participants knew any of the details about the meditations; we expected these instruction-naïve biases would predict higher partiality at later time-points (H1b: Bias).

#### Sensory Discriminability

Individual differences sensory discriminability – the ability to detect differences between sensory events within a given sensory modality – could plausibly allow some individuals to experience rewarding psychophysiological states more readily when using an anchor within modalities to which they are more sensitive. As each of the three experimental anchors engages a different sensory modality we predicted that modality-specific sensory discriminability would predict higher partiality for anchors within that modality (H2: Sensory discriminability).

#### Dispositional Variables

Dispositional personality and mindfulness variables were also expected to meaningfully predict preferences (H3: Personality). Conscientiousness predicts health-beneficial behaviours (Murray and Booth, 2015) thus higher Conscientiousness was expected to predict higher partiality as meditation has well-known health benefits. Openness was expected to predict higher partiality as these practices were novel to all our meditation-naïve participants. The affective volatility of Neuroticism is counter to the relaxed engagement promoted by contemplative practice thus it was expected to predict lower partiality. No directional predictions were made for Extraversion and Agreeableness. We expected that mindfulness would predict higher overall partiality. Mind-wandering was expected to predict lower partiality towards the meditations because mind-wandering is the conceptual opposite of mindfulness; as such, mind-wandering could make meditation feel difficult and reduce the overall ratings towards all three anchors.

#### Physiological Response

We expected that cardiac and respiratory experiences of rewarding psychophysiological states during practice would predict higher partiality. Specifically, we expected that decreases in heart-rate and increases in high-frequency heart-rate-variability would predict higher partiality (H4a and b, respectively). Lower HR and higher HF-HRV have been considered physiological markers of beneficial practice (Olex et al., 2013; Shearer et al., 2015). Similarly, we expected that decreases in respiration-rate would predict higher partiality (H4c).

## Materials and Methods

Questionnaires, stimuli, code, and practice instructions are shared on the Open Science Framework (Anderson and Farb, 2016). The raw data supporting the conclusions of this manuscript will be made available by the authors, without undue reservation, to any qualified researcher. This research was performed in accordance with the Declaration of Helsinki under the auspices of the Social Sciences, Humanities and Education Research Ethics Board at the University of Toronto, Mississauga campus.

### Participants and Procedure

Meditation-naïve undergraduates participated in exchange for course-credit or financial remuneration (20 CAD). In lieu of a power analysis, we planned to collect 100–130 participants, dependent on availability. In total 117 participants were recruited and 82 (70%) were retained for analysis: the investigator (TA) and 3 research assistants independently reviewed open-ended comprehension/compliance questions and agreed (Fleiss’ kappa = 0.365) that 35 participants did not comply with practice instructions (discussed in detail in section 5). In cases of disagreement between raters, TA further reviewed the response and made a final decision regarding inclusion/exclusion; all such decisions were made prior to data-analyses. The final sample was, on average, 20 years old (SD: 2.25) and 59% female; age and gender did not moderate results.

### Practice Instructions

Practice instructions were an unsupervised, manualised practice with an attention-anchor. Instructions were uniform across meditation conditions save for anchor modality descriptions: the somatosensory anchor was the sensation of breathing, the auditory anchor was a meaningless word-like phrase (“ay-lo-ra”) played through headphones during instructions only, and the visual anchor was a simple image (green circle) shown on-screen during instructions.

### Questionnaires

#### Subjective Partiality

**FIGURE 1:**
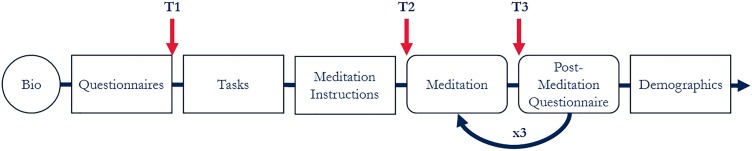
Experimental procedure. First, participants put on the BioHarness. Participants were then seated at a computer with dividers providing separation from other participants. They followed on-screen instructions for the remainder of the experiment. Participants completed computer-based questionnaires, then sensory discriminability and mind-wandering tasks. Participants read general meditation instructions, then anchor-specific instructions for three 10-min meditation practices (breath, phrase, and image). All participants engaged each of the meditation anchors in a random order and anchor-order did not moderate results. Partiality towards each anchor was collected at three time-points: before knowing anything about the meditations (T1: initial instruction-naïve biases), after reading the instructions but before practicing (T2: practice-naïve motivation), and after practicing with the anchor for 10 min (T3: post-practice partiality).

Participants were told they would experience three meditations with different anchors. Before participants read any meditation instructions, initial bias was assessed by asking participants “for each type of meditation please rate how much you expect to enjoy it” (Figure 1, T1). After reading the instructions and immediately before engaging each meditation motivation was assessed by asking participants “please indicate how motivated you presently feel to engage in the meditation” (Figure 1, T2). Following each 10-min practice participants indicated post-practice partiality (Figure 1, T3). The literature contained no standard measure of post-meditation partiality thus a novel questionnaire was developed (sample items: “I found the experience physically relaxing or restful,” “I found the meditation difficult”). All items were rated using 0 (“Not at all”) to 100 (“Very much”) continuous slider scales and partiality for a particular anchor was computed as the average of the 15 retained items (α = 0.92).

#### Dispositional Personality

The Big Five Inventory (BFI; McCrae and John, 1992) measures personality using the five-factor model. The original BFI contains 44 statements (“I see myself as someone who…”) that participants rate on a 5-point scale. In the present study all items were rated using 0–100 slider scales with nominal descriptors at 0 (“Strongly Disagree”), 50 (“Neutral”), and 100 (“Strongly Agree”) (Matejka et al., 2016). This alteration did not negatively impact reliability (Extraversion α = 0.87, Agreeableness α = 0.77, Conscientiousness α = 0.82, Neuroticism α = 0.83, and Openness α = 0.71).

#### Dispositional Mindfulness

Dispositional mindfulness was assessed through two scales: The Mindful Attention Awareness Scale (MAAS; Brown and Ryan, 2003; five-item short-form, Van Dam et al., 2010; Osman et al., 2015), and the Cognitive and Affective Mindfulness Scale Revised (CAMS-R; Feldman et al., 2006). MAAS and CAMS-R items were averaged into one “MIND” score: the MAAS reverse-scores constructs opposed to mindfulness, i.e., “automatic-ness,” and the CAMS-R measures positive aspects of mindfulness: attention regulation, present-minded awareness, and non-judgemental acceptance. To retain uniformity with the BFI, MAAS and CAMS-R items were rephrased to match the BFI style (“I see myself as someone who…”) and were rated using the same 0–100 slider scales, which did not negatively impact reliability (MIND α = 0.75, MAAS α = 0.73, and CAMS-R α = 0.67).

### Tasks

#### Behavioural Mind-Wandering

The Metronome Response Task (MRT) was used to behaviourally measure mind-wandering, which has been linked to response-variability when participants tap the steady beat of the MRT (Seli et al., 2013; Anderson et al., 2017). For further details see Supplementary Materials.

#### Sensory Discriminability

Sensory discriminability was measured by three psychophysical staircases in the three anchor modalities: somatosensory (vibration-detection), auditory (pitch-discrimination), and visual (colour-saturation discrimination). In each staircase procedure, participants were presented with a series of target stimuli and foil stimuli; their discrimination accuracy dynamically adjusted the stimuli in order to hone in on the smallest difference the individual could discern. For further details see Supplementary Materials.

### Physiological Recording

Participants wore a heart- and respiration-rate monitor for the duration of the experiment (BioHarness 3; Zephyr Technology, Annapolis, MD, United States). Heart-rate, high-frequency heart-rate-variability, and respiration-rate were recorded (Johnstone et al., 2012a,b).

## Results

### Data Analysis

Multilevel modelling was used to predict Partiality (Figure 1, T3), nested within participant (Enders and Tofighi, 2007), and model-fits are reported as Bayesian Information Criterion (BIC, Schwarz, 1978) and Akaike Information Criterion (AIC, Akaike, 1973). *A priori* stepwise linear regression used an intercept-only baseline model. Partiality existence (H1) was assessed by using Anchor-type as a predictor, Motivation (H1a) was tested by adding Motivation (Figure 1, T2), Bias (H1b) by adding Bias (Figure 1, T1), and Personality (H3) by adding dispositional personality and mindfulness variables.

Attrition from tasks was not anticipated thus modelling Sensory discriminability (H2) has been rearranged: 77 of 82 participants completed psychophysical staircases thus the model testing H2 was computed separately to avoid limiting model sample sizes. The best model attained above served as the base-model and the modality-specific sensory discriminability scores were added to test H2. Similarly for the mind-wandering component of our Personality hypothesis (H3), as only 62 of 82 participants completed the MRT it was computed separately as an extension to the original hypothesis (H3_MRT_).

#### Physiological Data Pre-processing

Heart-rate (HR), high-frequency heart-rate-variability (HF-HRV), and respiration-rate (RR) were recorded as markers of physiological response to meditation. ECG was recorded at 250 Hz, R-to-R intervals interpolated at 4 Hz, and HR filtered to 30–125 bpm. Following Berntson et al. (1997) two 4-min epochs were created by trimming the first and last minutes of practice periods in order to allow a buffer for participants to become engaged with each practice. Within-subjects baselines were calculated as averages of the medians of two 4-min epochs (questionnaire and task periods). HR change scores (ΔHR) are the percent difference between baseline and practice-epoch median. Respiration-rate was filtered to 6–30 breaths-per-minute and ΔRR change scores calculated in the same manner. HRVs were computed with Fourier transforms, HF band-power (0.15 to 0.4 Hz) was analysed, medians were log-transformed, and ΔHF-HRV change scores calculated.

The Zephyr BioHarness 3 fits under the clothing thus participants affixed it themselves. Unfortunately, 30–35% of cardiac data was problematic (not properly collected, intermittent device connectivity, excessive noise) thus these data-points were dropped (HR: 25, HF-HRV: 29, RR: 3). As with task analyses the models testing H4a-c were computed separately with the best model attained by the stepwise regression. Physiological response during both halves of practice showed considerable collinearity (ΔHR *r* = 0.87, ΔHF-HRV *r* = 0.82, and ΔRR *r* = 0.68) thus are not modelled together; the second-half recordings consistently captured more variance and are reported.

### Prevalence of Partiality

**FIGURE 2:**
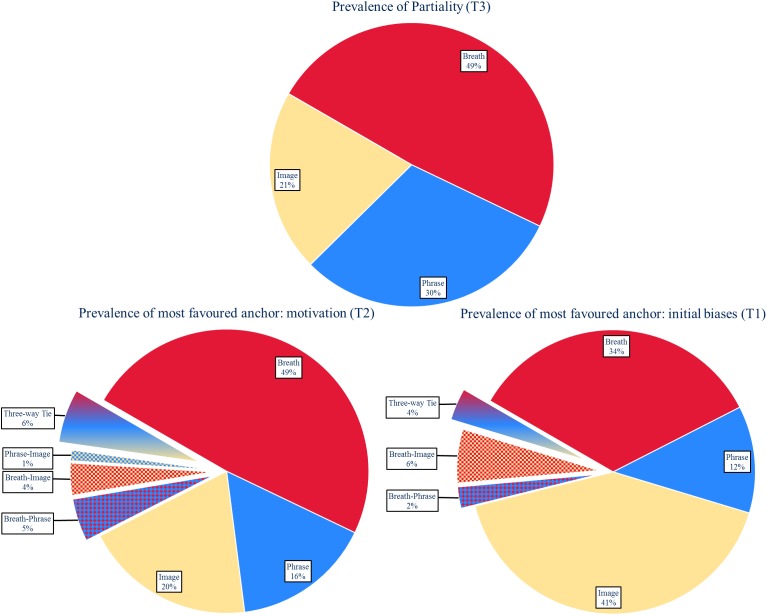
Distributions of partiality: differences in partiality were found throughout the experiment, confirming H1. T3 shows that 49% (95% CI [39 and 60%]) of participants favour the breath once given the opportunity to try the practice. While breath was the most favoured anchor in our sample, this anchor is not favoured by all participants, demonstrating that further investigation into anchors of attention that are not body-based are warranted.

Confirming Partiality existence (H1) individual differences were found in anchor partiality at every stage of the experiment (see Figure 2). The most-favoured anchor in instruction-naïve participants (T1) were Image (41 and 95% CI [30 and 53%]) followed closely by Breath (34 and 95% CI [23 and 45%]). After reading instructions, participants indicated motivation to engage each practice with the highest motivation being Breath (49 and 95% CI [39 and 60%]) followed by Image (20 and 95% CI [10 and 31%]). After practicing each meditation for 10 min the most-favoured anchor was Breath (49 and 95% CI [39 and 60%]) followed by Phrase (30 and 95% CI [20 and 42%]). Most favoured anchor changed at some point for 77% (95% CI [68 and 86%]) of participants and 56% (95% CI [46 and 68%]) of participants left the experiment partial to a different anchor than their instruction-naïve bias.

### Predicting Partiality

The intraclass correlation coefficient indicated within-participant partiality ratings were not independent (ICC = 0.294, Peugh, 2010). Motivation (H1a) was the best-fitting model demonstrating a large significant effect of motivation (β = 0.456, *p* < 0.0001, *r* = 0.52) with greater motivation predicting greater post-practice partiality. A moderate effect of anchor (β-Breath = 0.174, β-Phrase = 0.156, β-Image = −0.330, *p* < 0.0001, *r* = 0.38) reflected that, on average, participants preferred Breath (M: 62.63, SD: 1.93) and Phrase (M: 59.35, SD: 2.02) practices over the Image (M: 51.32, SD: 2.23) practice [Breath: *t*(81) = 4.407, *p* < 0.0001, Phrase: *t*(81) = 3.53, *p* < 0.001]. Counter to Bias (H1b), Sensory discriminability (H2), and Personality (H3 and H3_MRT_) models with additional predictors were not superior (see Tables 1, 2). Supporting H4a/b cardiac response predicted partiality (ΔHR: β = 0.185, *p* < 0.014, *r* = 0.24, ΔHF-HRV: β = 0.20, *p* < 0.009, *r* = 0.26), yet counter to H4c respiration did not (*p* = 0.526) (Table 3). As anchor did not predict cardiac responses (ΔHR: *p* = 0.69, ΔHF-HRV: *p* = 0.39) they were not pursued as mediators of the anchor-partiality relationship.

**Table 1:** Model estimates for partiality from hypothesised predictors.

	Intercept	Partiality Existence H1			Motivation H1a			Bias H1b			Personality H3		
Model BIC	695	683			**637**			642			671		
Model AIC	684	665			**616**			618			625		

**Predictor**		**β**	***SE***	***r***	**β**	***SE***	***r***	**β**	***SE***	***r***	**β**	***SE***	***r***

Anchor	Breath	0.25	0.11	0.36	0.17	0.09	0.38	0.17	0.10	0.38	0.18	0.10	0.37
	Phrase	0.08	0.11		0.16	0.09		0.15	0.10		0.14	0.10	
	Image	−0.34	0.11		−0.33	0.09		−0.32	0.10		−0.32	0.10	
Motivation					0.46	0.06	0.52	0.47	0.07	0.49	0.45	0.07	0.46
Bias								−0.03	0.06	0.04	−0.04	0.06	0.05
Mindfulness											0.11	0.09	0.13
Conscientiousness											−0.04	0.08	0.06
Openness											0.06	0.07	0.09
Neuroticism											−0.02	0.08	0.02
Extraversion											0.01	0.07	0.01
Agreeableness											0.05	0.07	0.08

*Bold indicates the best-fitting model AIC and BIC values.*

**Table 2:** Model estimates for partiality from tasks.

		Motivation H1a	Sensory discriminability H2			Motivation H1a	Personality H3_MRT_		
Subset N^∗^		77	77			62	62		
Model BIC		**562**	566			**498**	503		
Model AIC		**542**	542			**479**	481		

**Predictor**			**β**	***SE***	***r***		**β**	***SE***	***r***

Anchor	Breath		0.19	0.10	0.39		0.15	0.11	0.41
	Phrase		0.14	0.10			0.15	0.11	
	Image		−0.36	0.10			−0.42	0.11	
Motivation			0.45	0.06	0.54		0.49	0.07	0.54
Sensory discriminability			−0.08	0.06	0.12				
MRT variability							0.01	0.07	0.01

*^∗^To facilitate BIC/AIC comparisons H1a models reported in this table use only the participant subsets that successfully completed the tasks. Bold indicates the best-fitting model AIC and BIC values.*

**Table 3:** Model estimates for partiality from physiological responses to brief practice.

		Motivation H1a	ΔHR H4a			Motivation H1a	ΔHF-HRV H4b			Motivation H1a	ΔRR H4c		
Subset N^∗^		57	57			53	53			79	79		
Model BIC		457	**456**			427	**425**			**600**	606		
Model AIC		438	**434**			409	**404**			**580**	582		

**Predictor**			**β**	***SE***	***r***		**β**	***SE***	***r***		**β**	***SE***	***r***
Anchor	Breath		0.15	0.12	0.42		0.10	0.12	0.38		0.18	0.10	0.35
	Phrase		0.12	0.12			0.14	0.12			0.13	0.10	
	Image		−0.47	0.12			−0.43	0.12			−0.34	0.10	
Motivation			0.42	0.07	0.51		0.43	0.07	0.52		0.46	0.06	0.53
ΔHR			0.19	0.07	0.24								
ΔHF-HRV							0.20	0.08	0.26				
ΔRR											−0.04	0.06	0.05

*^∗^To facilitate BIC/AIC comparisons H1a models reported in this table use only the participant subsets with physiological data. Bold indicates the best-fitting model AIC and BIC values.*

#### Predicting Motivation and Bias

Motivation was uncovered as the primary predictor of post-practice partiality. While an interesting and expected finding, this result begs two questions: (1) what predicts motivation to engage a practice, and (2) what predicts initial instruction-naïve biases. These questions are explored in the Supplementary Materials: Motivation was predicted by anchor (β-Breath = 0.143, β-Phrase = −0.034, β-Image = −0.108, *p* < 0.0124, *r* = 0.26) and initial biases (β = 0.324, *p* < 0.0001, *r* = 0.44) whereas initial biases were predicted solely by anchor (β-Breath = 0.096, β-Phrase = −0.389, β-Image = 0.293, *p* < 0.0001, *r* = 0.37).

## Discussion

The present study investigated whether there exist differences in partiality towards meditative anchors of attention. Breath-anchored meditation is the most popular contemporary practice and is central to popular MBIs, but when asked before reading anything about the meditations (T1) only one third of participants expected to enjoy the breath most; more participants expected to enjoy an image-based practice. The lack of consensus was also present after reading instructions for and engaging three different anchors. While the breath anchor has popular appeal and 49% of the participants favoured the breath over the other two anchors tested in this study, these results demonstrate that partiality is not universal as the other 51% of participants were partial to auditory or visual anchors when given the choice. Focusing initial training exclusively on somatosensory anchors may create lost opportunities to incentivise early meditation training as some aspiring meditators may be more likely to establish and maintain self-directed practice habits if taught an auditory-phrase or visual-image practice. Offering alternate anchors is a tangible next-step that could be integrated into existing interventions.

While asking a potential meditator about anticipated favourites offers a starting point for improving engagement, reviewing instructions and offering actual experience informs which anchor is ultimately preferred and could be suggested for self-directed practice. Partiality often shifted after participants read the mediation instructions and shifted again after their brief practice engaging the three anchors thus both conceptual and experiential knowledge were informative. Indeed, it was surprising how flexible partiality was given that a mere 10 min of practice with each anchor was sufficient to shift opinions for 77% of participants and half the participants left the experiment partial to a different anchor than when entering. Initial instruction-naïve biases were not significant predictors of which anchor participants ultimately preferred; instead, motivation to engage the specific anchor was the major predictor, itself informed by reading anchor-specific meditation instructions. Given the changes seen after engaging each anchor and the mutability of partiality away from initial biases we encourage future studies to investigate the possible benefits of offering different anchors to participants. This study offered three options, but there are numerous other types of meditation not explored in this work that may have differentiable effects (e.g., body-scan, complex visualisations, guided meditations, loving-kindness, compassion, etc.). Much work still needs to be done exploring the outcomes of using different anchors and meditation styles as the underlying similarities, differences, and mechanisms are not yet clear.

One potential beneficial pathway of meditation is homeostasis-facilitation (Farb et al., 2015) thus understanding the different physiological states promoted by different anchors could help pair new meditators with individually adaptive practices. Breath-anchored practice promoted respiratory slowing, but respiration changes were unrelated to partiality; in contrast, no anchor uniquely promoted cardiac response, though individual cardiac responses were associated with partiality. Unfortunately, the technology we employed to monitor cardiac response was somewhat unreliable (30–35% data-loss). With this limitation in mind, and with the broader awareness that the physiological effects of different anchors have not been fully categorised, future studies should continue to explore anchor-specific physiological responses and how they inform practice-optimisation.

Our results did not support the hypothesis that sensory discriminability would lead to within-modality partiality. Sensory modalities parallel the literature on modality-specific “learning styles,” which have rich public appeal despite research showing no true effects (Kampwirth and Bates, 1980; Pashler et al., 2008; Willingham et al., 2015). Perhaps, as with “learning styles,” this hypothesis is best abandoned. Similarly, dispositional personality and mindfulness were not meaningfully predictive of partiality, which was best explained by motivation and anchor type. We did adjust the data-collection scales for our mindfulness measures so this finding needs to be replicated and we do believe that future work should continue to explore predictors that may be associated with partiality and treatment outcome.

### Limitations

Participants were screened for understanding of practice instructions and thirty-five participants (30%) were removed for comprehension/compliance issues. After reading the meditation instructions, participants were asked, “please describe the meditation you are about to do in your own words.” These reports were reviewed and participants that responded with a description that did not match the instructions were removed from analysis for non-comprehension. Likewise, after each meditation, participants were asked, “please feel free to add any personal comments about the experience here.” Many participants reported non-compliance and were thus removed. While any reduction in sample-size decreases power and promotes survivorship-bias, it is nevertheless conceptually important for contextualising the findings that we include only participants that understood and followed meditation instructions. Furthermore, this 30% parallels the 30% drop out rates in MBSR courses (Crane and Williams, 2010). We recommend including open-ended qualitative comprehension/compliance checks anywhere meditation is taught or researched.

### Future Directions

This study reveals research-gaps concerning the relationships between partiality, practice-maintenance, and potential benefits of meditation. While we raise these issues, the present study was not designed to address them as it lacked a longitudinal practice-maintenance component. We believe that facilitating practice-maintenance begins by investigating barriers that may prevent participants from establishing at-home practice, one of which may be individual differences in partiality towards limited anchor options. Our interventions were not intended to initiate participants into an ongoing practice, and multi-session, hands-on MBI training is vastly different than our single-session, instruction-based training. Indeed, this study raises issues with current training, but cannot, in itself, solve them. Instead, we provide evidence for the existence of variability in partiality and call for future work to find better ways of incorporating these individual differences into MBIs that aim to promote at-home practice. Dismantling studies could investigate adherence differences in MBI cohorts, comparing MBI-as-usual to an MBI supplemented with additional anchor options (e.g., phrase and image). We have shared our study materials to facilitate this future work (Anderson and Farb, 2016).

Future studies should also investigate the circumstances precipitating practice-cessation (Vuckovich, 2010; Anderson et al., 2016). Research should measure partiality, practice-maintenance, and benefits in the weeks, months, and years following initial meditation training from various sources (Farb et al., 2018). Measuring practice-maintenance and cessation would not be difficult and more emphasis should be put on automating very-long-term follow-up of self-directed practice. We recommend increasing post-training contact using email-automation: sending short digital questionnaires at regular intervals and collecting data indefinitely. Probing participants every six- to twelve-months about if and how often they practice would be an easy and extremely effective next step for answering practice-maintenance questions, including whether and which contemplative benefits are predicated on continued practice (Grossman et al., 2004; Farb et al., 2018).

## Conclusion

Research into various meditation anchors should continue. New meditators show mutable partiality towards different anchors that can be predicted by motivation and changed by brief experience: meditation teachers should offer anchors in different sensory modalities, having novices experience a couple different practices before picking one. Meditation teachers and researchers should ensure that new meditators demonstrate instruction-comprehension as without proper understanding and implementation there is little hope of establishing beneficial practice. Even when self-directed practice is established we lack an understanding of practice-cessation. We should encourage very-long-term follow-up of practice-maintenance trajectories to measure how self-directed practice relates to benefits and to understand when and why practice is ceased. Such a picture of real-world practice over months and years would allow us to adapt practices for the influx of people seeking health and well-being through contemplative practice.

## Author Contributions

All authors contributed to conception and design of the study, considerable manuscript revisions, and approved the submitted version. TA performed the statistical analysis and wrote the first draft of the manuscript.

## Conflict of Interest Statement

The authors declare that the research was conducted in the absence of any commercial or financial relationships that could be construed as a potential conflict of interest. The reviewer GM and handling Editor declared their shared affiliation at the time of the review.
