# Supplementary material for: Personalising Practice Using Preferences for Meditation Anchor Modality
Source: Front Psychol. 2018 Dec 11;9:2521. doi: 10.3389/fpsyg.2018.02521 (PMC6298414; doi:10.3389/fpsyg.2018.02521)
Supplement: Supplementary file 1 [file Data_Sheet_1.docx]

# Supplemental Materials

## 2. Methods

Questionnaires, stimuli, code, and practice instructions shared on the Open Science Framework (Anderson and Farb, 2016).

## 2.4 Tasks

### 2.4.1 Behavioural mind-wandering

The Metronome Response Task (MRT) was used to behaviourally measure mind-wandering, which has been linked to response-variability when participants tap the steady beat of the MRT (Anderson et al., 2017; Seli et al., 2013). As in Seli et al. (2013) MRT trials were as follows: 650 ms of silence followed by a tone lasting 75 ms followed by another 575 ms of silence, resulting in a total trial duration of 1300 ms. In the original, total MRT duration was 20min, but due to time constraints participants instead completed a 1 min practice block followed by a 5 min MRT block. Response time variability was computed as in Seli et al. (2013): participants missing more than 10% of trials dropped, missed trials dropped, and scores log-transformed.

### 2.4.2 Sensory discriminability

Three psychophysical staircases measured sensory discriminability in the three anchor modalities: somatosensory (vibration-detection), auditory (pitch-discrimination), and visual (colour-saturation discrimination). Sensory discriminability was measured using three modalities of psychophysical staircase programmed in PsychoPy (Peirce, 2007, 2009). As per Garcı́a-Pérez (1998) the staircases followed a 2-Alternative Forced-Choice (2AFC) 1-Up/3-Down procedure with 10 reversals. The final 6 reversals were used to calculate individual Just-Noticeable Difference (JND) thresholds, which were Winsorized to two standard deviations and log-transformed. Failure to discriminate was rare (9% of staircases); technical issues prevented five participants from completing any staircases.

Somatosensory-discrimination involved indicating which of two intervals contained a vibrating stimulus (vs non-vibrating), an index of bodily awareness (Mirams et al., 2013). Auditory-discrimination involved indicating which of two intervals contained a 440 Hz reference tone (vs foil tone, higher or lower in pitch). Visual-discrimination involved indicating which of two intervals contained the reference image (a dim green circle vs foil colour, more or less saturated.

At the start of each staircase participants were presented with the reference stimulus (vibration, tone, circle) and then asked to judge which of two sequential stimuli matched the reference stimulus. Every trial contained a "target" stimulus matching the reference stimulus and was contrasted with a non-matching "foil" stimulus. The staircase procedure began with trials in which the difference between the target and foil was large. Each incorrect trial resulted in the difference between target and foil increasing one step (1-Up); three consecutive correct trials resulted in the difference between target and foil decreasing one step (3-Down). A "reversal" - a change in direction from increasing difference to decreasing or vice versa - reflects a crossing of the participants' Just Noticeable Difference threshold (JND). For each two reversals the step size decreased by half, narrowing around the specific boundary of the JND threshold. Ten reversals were collected, the last six were averaged, then Winsorized and log-transformed, creating JND scores.

To improve data quality within-task screening was employed for the staircases. If participants failed to evince discrimination between target and foil stimuli at the maximally distinct thresholds (MDT) the instructions and reference were reviewed and the staircase began anew. If participants were again unable to demonstrate stimulus discrimination at MDT the task terminated early and the participant’s JND for the task was treated as missing data. Failure to discriminate above threshold was rare and occurred in 33 staircases (9% of all staircases: 10 vibration, 11 auditory, and 11 visual). Five participants could not complete any of the staircases due to technical issues with the testing computers.

In somatosensory-discrimination participants were asked to indicate which of the two intervals contained a vibrating stimulus, an index of bodily awareness (Mirams et al., 2013), using a bone-conductor hearing-aid (Connevans Ltd., United Kingdom) and told to hold the device lightly yet firmly between the thumb and index-finger of their left hand. Intensity of vibration increased or decreased until ten staircase reversals were collected and the threshold was determined (MDT: maximum-amplitude-vibrating vs non-vibrating). In the case of auditory-discrimination participants were asked to indicate which of the two intervals contained a 440 Hz sine-wave tone. Participants wore headphones and foil tones were randomly higher or lower in pitch (MDT: 4 times or ¼ the reference: 1760 Hz - 110 Hz). In the case of visual-discrimination participants were asked to indicate which of the two intervals contained an on-screen circle filled with a dim green (RGB values (0%, 50%, 0%)). Foil colours were randomly more or less saturated (MDT: white or black circle).

## 3. Results

### 3.3.1 Predicting motivation and bias

Motivation was uncovered as the primary predictor of post-practice partiality. While an interesting and expected finding (H1a) this result begs the questions: 1) what predicts motivation and 2) what predicts instruction-naïve biases. We explored these questions using a series of models of the same form as those planned for partiality, substituting motivation and initial biases as the outcome variables. While based on planned models for partiality, the application of these models to these outcome variables was not planned a priori thus these analysis should be interpreted as exploratory.

#### 3.3.1.1 Predicting motivation

Within-participant motivation levels across anchor types were highly related (ICC=0.542). The model with the best BIC supported H1b by showing a significant moderate effect of initial biases (β=0.324, p<0.0001, r=0.44) and of anchor (β-Breath=0.143, β-Phrase=-0.034, β-Image=-0.108, p<0.0124, r=0.26), revealing that after reading meditation instructions participants were, on average, more motivated to engage the Breath (M: 69.15, SD: 23.02) practice over the Phrase (M: 61.80, SD: 23.49) practice (t(81)=3.056, p<0.003); the Image (M: 65.04, SD: 18.78) practice was in-between. Counter to H2 models including sensory discriminability were not superior. The model with the best AIC supported H3 as superior with higher responses on Mindfulness, Conscientiousness, Openness, Neuroticism, Extraversion, and Agreeableness predicting higher motivation (β-M: 0.221, β-C: 0.029, β-O: 0.189, β-N: 0.214, β-E: 0.073, β-A: 0.053). H3_MRT_ was not supported.

| Table S1. Model estimates for motivation.   \|  \| Intercept \| H1 \| \| \| H1b \| \| \| H3 \| \| \| \| --- \| --- \| --- \| --- \| --- \| --- \| --- \| --- \| --- \| --- \| --- \| \| Model BIC \| 646 \| 647 \| \| \| **617** \| \| \| 634 \| \| \| \| Model AIC \| 635 \| 629 \| \| \| 596 \| \| \| **592** \| \| \| \| Predictor \|  \| β \| SE \| r \| β \| SE \| r \| β \| SE \| r \| \| Anchor \| Breath \| 0.17 \| 0.11 \| 0.25 \| 0.14 \| 0.10 \| 0.26 \| 0.14 \| 0.09 \| 0.25 \| \|  \| Phrase \| -0.16 \| 0.11 \| -0.03 \| 0.10 \| -0.04 \| 0.10 \| \|  \| Image \| -0.01 \| 0.11 \| -0.11 \| 0.10 \| -0.10 \| 0.10 \| \| Bias \|  \|  \|  \|  \| 0.32 \| 0.05 \| 0.44 \| 0.31 \| 0.05 \| 0.41 \| \| Mindfulness \|  \|  \|  \|  \|  \|  \|  \| 0.22 \| 0.10 \| 0.24 \| \| Conscientiousness \| \|  \|  \|  \|  \|  \|  \| 0.03 \| 0.09 \| 0.04 \| \| Openness \|  \|  \|  \|  \|  \|  \|  \| 0.19 \| 0.08 \| 0.28 \| \| Neuroticism \|  \|  \|  \|  \|  \|  \|  \| 0.21 \| 0.09 \| 0.26 \| \| Extraversion \|  \|  \|  \|  \|  \|  \|  \| 0.07 \| 0.08 \| 0.11 \| \| Agreeableness \| \|  \|  \|  \|  \|  \|  \| 0.05 \| 0.08 \| 0.07 \| |
| --- | --- | --- | --- | --- | --- | --- | --- | --- | --- | --- | --- | --- | --- | --- | --- | --- | --- | --- | --- | --- | --- | --- | --- | --- | --- | --- | --- | --- | --- | --- | --- | --- | --- | --- | --- | --- | --- | --- | --- | --- | --- | --- | --- | --- | --- | --- | --- | --- | --- | --- | --- | --- | --- | --- | --- | --- | --- | --- | --- | --- | --- | --- | --- | --- | --- | --- | --- | --- | --- | --- | --- | --- | --- | --- | --- | --- | --- | --- | --- | --- | --- | --- | --- | --- | --- | --- | --- | --- | --- | --- | --- | --- | --- | --- | --- | --- | --- | --- | --- | --- | --- | --- | --- | --- | --- | --- | --- | --- | --- | --- | --- | --- | --- | --- | --- | --- | --- | --- | --- | --- | --- | --- | --- | --- | --- | --- | --- | --- | --- | --- | --- | --- | --- | --- | --- | --- | --- | --- | --- | --- | --- | --- | --- | --- | --- | --- | --- | --- |

| Table S2. Model estimates for motivation from tasks.   \|  \|  \| H1b \| H2 \| \| \| H1b \| H3_MRT_ \| \| \| \| --- \| --- \| --- \| --- \| --- \| --- \| --- \| --- \| --- \| --- \| \| Subset N* \|  \| 77 \| 77 \| \| \| 62 \| 62 \| \| \| \| Model BIC \|  \| **549** \| 555 \| \| \| **471** \| 475 \| \| \| \| Model AIC \|  \| **529** \| 531 \| \| \| **452** \| 453 \| \| \| \| Predictor \|  \|  \| β \| SE \| r \|  \| β \| SE \| r \| \| Anchor \| Breath \|  \| 0.12 \| 0.11 \| 0.29 \|  \| 0.16 \| 0.11 \| 0.27 \| \|  \| Phrase \|  \| -0.11 \| 0.11 \|  \| -0.04 \| 0.11 \| \|  \| Image \|  \| -0.13 \| 0.11 \|  \| -0.10 \| 0.11 \| \| Bias \|  \|  \| 0.35 \| 0.06 \| 0.47 \|  \| 0.30 \| 0.06 \| 0.41 \| \| JND Threshold \| \|  \| -0.02 \| 0.05 \| 0.03 \|  \|  \|  \|  \| \| MRT Variability \| \|  \|  \|  \|  \|  \| 0.10 \| 0.09 \| 0.15 \|   **To facilitate BIC/AIC comparisons H1b models reported in this table use only the participant subsets that successfully completed the tasks.* |
| --- | --- | --- | --- | --- | --- | --- | --- | --- | --- | --- | --- | --- | --- | --- | --- | --- | --- | --- | --- | --- | --- | --- | --- | --- | --- | --- | --- | --- | --- | --- | --- | --- | --- | --- | --- | --- | --- | --- | --- | --- | --- | --- | --- | --- | --- | --- | --- | --- | --- | --- | --- | --- | --- | --- | --- | --- | --- | --- | --- | --- | --- | --- | --- | --- | --- | --- | --- | --- | --- | --- | --- | --- | --- | --- | --- | --- | --- | --- | --- | --- | --- | --- | --- | --- | --- | --- | --- | --- | --- | --- | --- | --- | --- | --- | --- | --- | --- | --- | --- | --- | --- | --- | --- | --- | --- | --- |

#### 3.3.1.2 Predicting initial biases

Initial biases were much less predictable (ICC=0.118). The model with the best BIC supported H1 by showing a significant effect only for anchor (β-Breath=0.096, β-Phrase=-0.389, β-Image=0.293, p<0.0001, r=0.37) reflecting, on the average, more positive initial instruction-naïve biases in favour of the Image (M: 70.0, SD: 19.50) and Breath (M: 65.66, SD: 22.85) practices over the Phrase (M: 54.99, SD: 21.01) practice (Image: t(81)=5.19, p<0.00001, Breath: t(81)=3.62, p<0.001). H2 was not supported. The model with the best AIC supported H3 as superior with higher responses on Mindfulness, Openness, Neuroticism, Extraversion, and Agreeableness predicting higher Bias and lower Conscientiousness predicting more favourable initial biases (β-M: 0.237, β-C: -0.214, β-O: 0.149, β-N: 0.174, β-E: 0.081, β-A: 0.175). H3_MRT_ was not supported.

| Table S3. Model estimates for initial biases.   \|  \| Intercept \| H1 \| \| \| H3 \| \| \| \| --- \| --- \| --- \| --- \| --- \| --- \| --- \| --- \| \| Model BIC \| 710 \| **697** \| \| \| 708 \| \| \| \| Model AIC \| 700 \| 679 \| \| \| **670** \| \| \| \| Predictor \|  \| β \| SE \| r \| β \| SE \| r \| \| Anchor \| Breath \| 0.10 \| 0.11 \| 0.37 \| 0.10 \| 0.10 \| 0.37 \| \|  \| Phrase \| -0.39 \| 0.11 \| -0.39 \| 0.10 \| \|  \| Image \| 0.29 \| 0.11 \| 0.29 \| 0.10 \| \| Mindfulness \|  \|  \|  \|  \| 0.24 \| 0.09 \| 0.30 \| \| Conscientiousness \| \|  \|  \|  \| -0.21 \| 0.08 \| 0.30 \| \| Openness \|  \|  \|  \|  \| 0.15 \| 0.07 \| 0.26 \| \| Neuroticism \|  \|  \|  \|  \| 0.17 \| 0.08 \| 0.25 \| \| Extraversion \|  \|  \|  \|  \| 0.08 \| 0.07 \| 0.14 \| \| Agreeableness \| \|  \|  \|  \| 0.18 \| 0.07 \| 0.28 \| |
| --- | --- | --- | --- | --- | --- | --- | --- | --- | --- | --- | --- | --- | --- | --- | --- | --- | --- | --- | --- | --- | --- | --- | --- | --- | --- | --- | --- | --- | --- | --- | --- | --- | --- | --- | --- | --- | --- | --- | --- | --- | --- | --- | --- | --- | --- | --- | --- | --- | --- | --- | --- | --- | --- | --- | --- | --- | --- | --- | --- | --- | --- | --- | --- | --- | --- | --- | --- | --- | --- | --- | --- | --- | --- | --- | --- | --- | --- | --- | --- | --- | --- | --- | --- | --- | --- | --- | --- | --- | --- | --- | --- | --- | --- | --- | --- | --- | --- | --- | --- | --- |

| Table S4. Model estimates for initial biases from tasks.   \|  \|  \| H1 \| H2 \| \| \| H1 \| H3_MRT_ \| \| \| \| --- \| --- \| --- \| --- \| --- \| --- \| --- \| --- \| --- \| --- \| \| Subset N \|  \| 77 \| 77 \| \| \| 62 \| 62 \| \| \| \| Model BIC \|  \| **612** \| 617 \| \| \| **532** \| 536 \| \| \| \| Model AIC \|  \| **595** \| 596 \| \| \| **516** \| 517 \| \| \| \| Predictor \|  \|  \| β \| SE \| r \|  \| β \| SE \| r \| \| Anchor \| Breath \|  \| 0.09 \| 0.11 \| 0.35 \|  \| 0.11 \| 0.12 \| 0.36 \| \|  \| Phrase \|  \| -0.35 \| 0.12 \|  \| -0.40 \| 0.12 \| \|  \| Image \|  \| 0.26 \| 0.12 \|  \| 0.21 \| 0.12 \| \| JND Threshold \| \|  \| -0.04 \| 0.07 \| 0.05 \|  \|  \|  \|  \| \| MRT Variability \| \|  \|  \|  \|  \|  \| 0.10 \| 0.08 \| 0.15 \|   **To facilitate BIC/AIC comparisons H1 models reported in this table use only the participant subsets that successfully completed the tasks.* |
| --- | --- | --- | --- | --- | --- | --- | --- | --- | --- | --- | --- | --- | --- | --- | --- | --- | --- | --- | --- | --- | --- | --- | --- | --- | --- | --- | --- | --- | --- | --- | --- | --- | --- | --- | --- | --- | --- | --- | --- | --- | --- | --- | --- | --- | --- | --- | --- | --- | --- | --- | --- | --- | --- | --- | --- | --- | --- | --- | --- | --- | --- | --- | --- | --- | --- | --- | --- | --- | --- | --- | --- | --- | --- | --- | --- | --- | --- | --- | --- | --- | --- | --- | --- | --- | --- | --- | --- | --- | --- | --- | --- | --- | --- | --- | --- | --- |

## 4. Supplemental discussion

Dispositional personality and mindfulness show a less clear relationships with initial biases and pre-practice motivation. Model comparisons showed lower AIC when dispositional traits were included yet BIC was lower for more concise models. As the additional variables offer little theoretically interpretable nuance and more variance was explained by simpler predictors - anchor-type and initial biases predicted motivation while anchor type alone predicted biases - we suggest that it may not be meaningful to retain dispositional traits (Dziak et al., 2015).

# Supplemental References

Anderson, T., and Farb, N. (2016). Personalizing Practice using Preferences for Meditation Anchor Modality. *OSF*. doi:None.

Anderson, T., Lin, H., and Petranker, R. (2017). Replication of Seli et al 2013 “Wandering Minds and Wavering Rhythms.” *Open Sci. Framew.* doi:None.

Dziak, J. J., Coffman, D. L., Lanza, S. T., and Li, R. (2015). Sensitivity and specificity of information criteria. PeerJ PrePrints doi:10.7287/peerj.preprints.1103v2.

Garcı́a-Pérez, M. A. (1998). Forced-choice staircases with fixed step sizes: asymptotic and small-sample properties. *Vision Res.* 38, 1861–1881. doi:10.1016/S0042-6989(97)00340-4.

Mirams L., Poliakoff E., Brown R.J., and Lloyd D.M. (2013). Brief body-scan meditation practice improves somatosensory perceptual decision making. *Conscious. Cogn.* 22, 348–359. doi:10.1016/j.concog.2012.07.009.

Peirce, J. W. (2007). PsychoPy—Psychophysics software in Python. *J. Neurosci. Methods* 162, 8–13. doi:10.1016/j.jneumeth.2006.11.017.

Peirce, J. W. (2009). Generating Stimuli for Neuroscience Using PsychoPy. *Front. Neuroinformatics* 2. doi:10.3389/neuro.11.010.2008.

Seli, P., Cheyne, J. A., and Smilek, D. (2013). Wandering minds and wavering rhythms: Linking mind wandering and behavioral variability. *J. Exp. Psychol. Hum. Percept. Perform.* 39, 1–5. doi:10.1037/a0030954.
